# Effectiveness of salvage radiotherapy for superficial esophageal Cancer after non-curative endoscopic resection

**DOI:** 10.1186/s13014-020-01582-8

**Published:** 2020-06-01

**Authors:** Ikuno Nishibuchi, Yuji Murakami, Yoshinori Adachi, Nobuki Imano, Yuki Takeuchi, Ippei Tkahashi, Tomoki Kimura, Yuji Urabe, Shiro Oka, Shinji Tanaka, Yasushi Nagata

**Affiliations:** 1 Department of Radiation Oncology, Graduate School of Biomedical and Health Sciences, Hiroshima University, 1-2-3 Kasumi, Minami-ku, Hiroshima, 734-8551 Japan; 2 Department of Regeneration and Medicine Medical Center for Translation and Clinical Research, Hiroshima University Hospital, 1-2-3 Kasumi, Minami-ku, Hiroshima, 734-8551 Japan; 3 Department of Gastroenterology and Metabolism, Institute of Biomedical and Health Sciences, Graduate School of Biomedical and Health Sciences, Hiroshima University, 1-2-3 Kasumi, Minami-ku, Hiroshima, 734-8551 Japan; 4 Department of Endoscopy, Hiroshima University Hospital, 1-2-3 Kasumi, Minami-ku, Hiroshima, 734-8551 Japan

**Keywords:** Superficial esophageal cancer, Endoscopic resection, Radiotherapy, Elective nodal irradiation

## Abstract

**Background:**

Endoscopic resection is widely used as an effective treatment for superficial esophageal cancer. However, the risk of lymph node metastasis increases in cases of muscularis mucosae or deeper invasion, for which additional treatment such as radiotherapy or surgery is required. Accordingly, the current study investigated the efficacy and toxicity of salvage radiotherapy after non-curative endoscopic resection as an organ preservation strategy.

**Methods:**

We retrospectively reviewed 37 esophageal cancer patients who received salvage radiotherapy after non-curative endoscopic resection. The pathological invasion depths were the muscularis mucosae, submucosal layer, and muscularis propria in 14, 22, and one patient, respectively. All patients received external beam radiotherapy. Among them, eight received intraluminal brachytherapy following external beam radiotherapy. Elective nodal irradiation was administered to all patients. Twenty-five patients received concurrent platinum and fluorouracil-based chemotherapy.

**Results:**

The median follow-up time was 74 months (range: 3–212). The 5-year progression-free survival and overall survival rates were 64 and 78%, respectively. No local or regional lymph node recurrence was observed. The causes of death included esophageal cancer in one patient, metachronous esophageal cancer in one patient, other malignancies in eight patients, and other causes in six patients. Late cardiac toxicities ≥ grade 3 were observed in six patients, one of whom died of arrhythmia**.**

**Conclusions:**

Salvage radiotherapy after non-curative esophageal endoscopic resection is an effective treatment as an organ preservation strategy. Although muscularis mucosae and submucosal cancer have a high risk of lymph node metastasis, our results suggest that elective nodal irradiation contributes to reduced regional node metastases.

## Background

Recently, the number of patients with superficial esophageal cancer has tended to increase due to the development of endoscopic equipment [1]. The risk of lymph node metastasis in tumors confined to the epithelium and lamina propria mucosae is extremely low. Endoscopic resection (ER), including endoscopic mucosal resection (EMR) or endoscopic submucosal dissection (ESD), is recommended as standard therapy for these patients, because of the high local tumor control rate and minimal invasion. However, the risk of lymph node metastasis increases in cases with muscularis mucosae (MM) or deeper invasion. Lymph node metastasis occurs in 10–20% of MM or upper submucosal layer (SM1) invasion and 40–60% of the middle submucosal layer (SM2) or lower submucosal layer (SM3) invasion [2–7]. Careful observation is sometimes selected for cases of MM invasion with negative margins and no lymphovascular invasion (LVI). However, additional treatment is required in cases of MM invasion with positive margins or LVI or SM invasion. Although esophagectomy with lymph node dissection is considered standard therapy in these cases, radical surgery is highly invasive and related to increased morbidity and mortality [8–10].

Until 2002, our institution commonly performed intraluminal brachytherapy (IBT) combined with external beam radiotherapy (EBRT) for the treatment of superficial esophageal cancer. We previously reported the long-term treatment results in patients with superficial esophageal cancer who received this treatment [11]. Favorable treatment outcomes in mucosal cancer were achieved in that study. In addition, recent studies reported a comparable outcome of definitive concurrent chemoradiotherapy (CCRT) for superficial esophageal cancer to that of surgery [12–14]. Radiotherapy is less invasive than surgery and has an advantage as an organ preservation method. However, little is known about the safety and efficacy of salvage radiotherapy (RT) after EMR or ESD [15, 16]. Therefore, this study retrospectively evaluated the efficacy and toxicity of salvage RT as an organ preservation strategy for superficial esophageal cancer with MM or deeper invasion after non-curative EMR or ESD.

## Methods

### Patient and tumor characteristics

Thirty-seven patients with esophageal cancer treated with ER followed by RT with curative intent at Hiroshima University between 2000 and 2014 were eligible for this analysis. One patient who was lost to follow-up just after treatment was excluded from the analysis. The patient and tumor characteristics are summarized in Table 1. The median age of the 34 male and three female patients was 67 years (range: 53–85). Double cancers were observed in 16 patients. Among them, five had concurrent double cancer. Nine patients had a history of heart diseases, including hypertension in four patients, ischemic heart disease (IHD) in three patients, hypertension and IHD in one patient, and dilated cardiomyopathy in one patient. All patients had squamous cell carcinoma. The Nodal stage was determined by using computed tomography (CT) until 2010 and by using CT and positron emission tomography (PET)-CT from 2011. PET-CT was performed in 11 patients. The circumference of tumor was less than three-quarters in 25 patients, equal to or more than three-quarters in four patients, entire in four patients, and unknown in four patients. Twenty-two patients received EMR and 15 patients received ESD. The pathological invasion depths were the MM in 14 patients, SM in 22 patients, and MP in one patient. For patients with MM invasion, additional RT was administered in cases with a positive margin, LVI, or high risk of lymph node metastasis as judged by the gastroenterologists. Written and informed consent for RT was obtained from each patient before treatment. This retrospective analysis was approved by the institutional review board.

**Table 1:** Patient and tumor characteristics

Characteristics	No. of patients (%)
Age (years)	
Range	53–85
Median	67
Sex	
Male	34 (92)
Female	3 (8)
Performance status score	
0–1	34 (92)
2–3	3 (8)
History of double cancer	
Yes	16 (43)
No	21 (57)
History of heart disease	
Yes	9 (24)
No	28 (76)
Histology	
Squamous cell carcinoma	37 (100)
Tumor location	
Cervical	1 (3)
Upper thoracic	5 (13)
Middle thoracic	23 (62)
Lower thoracic or Esophagogastric junction	8 (22)
Tumor length (cm)	
Range	0.8–10
Median	3
Circumference	
< 3/4	25 (67)
≥ 3/4	4 (11)
Entire	4 (11)
Unknown	4 (11)
Methods of endoscopic resection	
EMR	22 (59)
ESD	15 (41)
Depth of pathological invasion	
Muscularis mucosae	14 (38)
Submucosal layer	22 (59)
Muscularis propria	1 (3)
Resection margin	
Negative	10 (27)
Positive	17 (46)
Non-assessable	6 (16)
Unknown	4 (11)

*EMR* endoscopic mucosal resection, *ESD* endoscopic submucosal dissection

### Treatment

#### Radiotherapy

Before performing a planning CT, metallic clips were placed endoscopically to indicate the excision region. EBRT was performed using a megavoltage photon beam (6–18 MV). Elective nodal irradiation (ENI) was administered to all patients and boost irradiation was performed after ENI. The clinical target volume (CTV) for ENI according to the primary tumor sites were cervical, supraclavicular, and upper mediastinal lymph nodes (LNs) for cervical tumors; supraclavicular, upper mediastinal, and subcranial LNs for upper thoracic tumors; upper to lower mediastinal and perigastric LNs for middle thoracic or lower thoracic tumors; and middle to lower mediastinal, perigastric and celiac artery LNs for esophagogastric junction tumors. Three-dimensional RT planning was performed for all the patients. ENI was performed by using anterior- posterior opposing beams in 20 patients, multi-portal beams in 16 patients and intensity-modulated radiation therapy (IMRT) in one patient. The IBT boost was used in combination with EBRT in eight patients, until 2002.

##### EBRT and IBT combination

IBT was performed using the high-dose-rate iridium-192 remote after loading system. A double-balloon applicator was used for IBT. The outer diameter of the applicator was 20 mm. The prescribed dose was calculated at a depth of 5 mm from the surface of the esophageal mucosa. Irradiation doses of EBRT were 54 Gy/27 fractions in cases of MM or SM1 invasion and 60 Gy/30 fractions in case of SM2 or SM3 invasion. The median ENI dose was 44 Gy/22 fractions (range: 44–45 Gy). The IBT boost was generally performed immediately after EBRT and the dose was 10 Gy/4 fractions. An excision region with a 2-cm longitudinal margin was irradiated.

##### EBRT alone

Twenty-nine patients were treated by EBRT alone. The median ENI dose was 40 Gy/20 fractions (range: 40–48 Gy) and the median total dose was 60 Gy/30 fractions (range: 50.4–66 Gy). An accelerated hyper-fractionation (AHF) schedule was used in two patients. CTV for boost was defined as an excision region with a 2 cm margin in the longitudinal direction.

#### Chemotherapy

Twenty-five patients received concurrent chemotherapy. The RT procedure was EBRT alone in 24 patients. The selection of chemotherapeutic regimen and reduction of chemotherapeutic dosages were determined according to the protocol at that time and the clinician’s judgment. The chemotherapeutic regimens were as follows: cisplatin/fluorouracil (5FU) in 14 patients, nedaplatin/5FU in 10 patients, and carboplatin/5FU in one patient.

### Analysis

The clinical data were updated in April 2020. Overall survival (OS) was defined as the time from the initiation of RT to death from any cause. Progression-free survival (PFS) was defined as the time from the initiation of RT to disease progression, death for any reason, or diagnosis of esophageal metachronous cancer. Esophageal metachronous cancer was defined as the secondary cancer detected in a different site from primary lesion after RT by endoscopy and was not included in local failure in this analysis. The Kaplan-Meier method was used to calculate survival rates. Log-rank tests were used to compare survival curves in univariate analysis. Comparison of data was analyzed by Fisher’s exact test. *P*-values < 0.05 were considered to indicate statistical significance. The Common Terminology Criteria for Adverse Events, version 4.0 (CTCAE v. 4.0) were used to assess toxicities. Acute and late toxicities were defined as events that occurred within or after three months from RT initiation, respectively.

## Results

### Survival and failure patterns

**Fig. 1:**
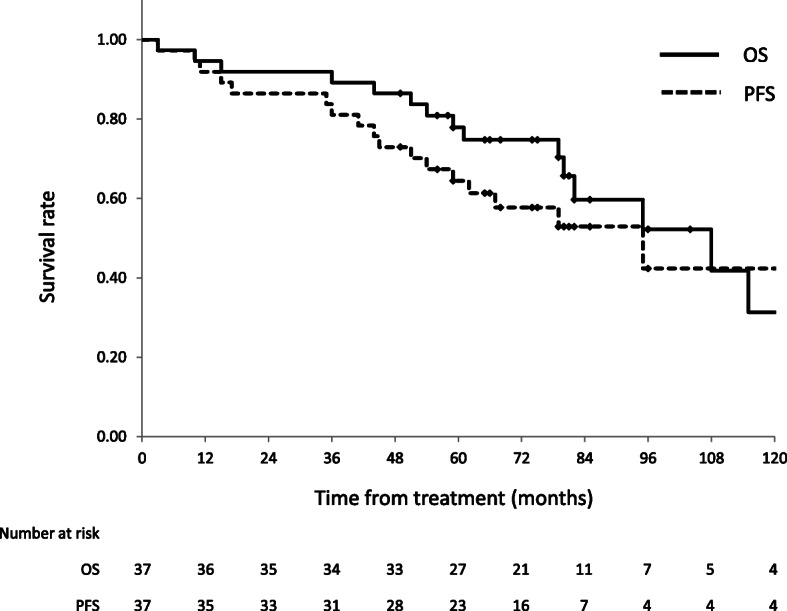
OS and PFS rates for all patients. The 5-year OS and PFS rates for all patients were 78% (95% CI, 64–91%) and 64% (95% CI, 49–80%), respectively. OS: overall survival, PFS: progression-free survival; CI: confidence interval

**Fig. 2:**
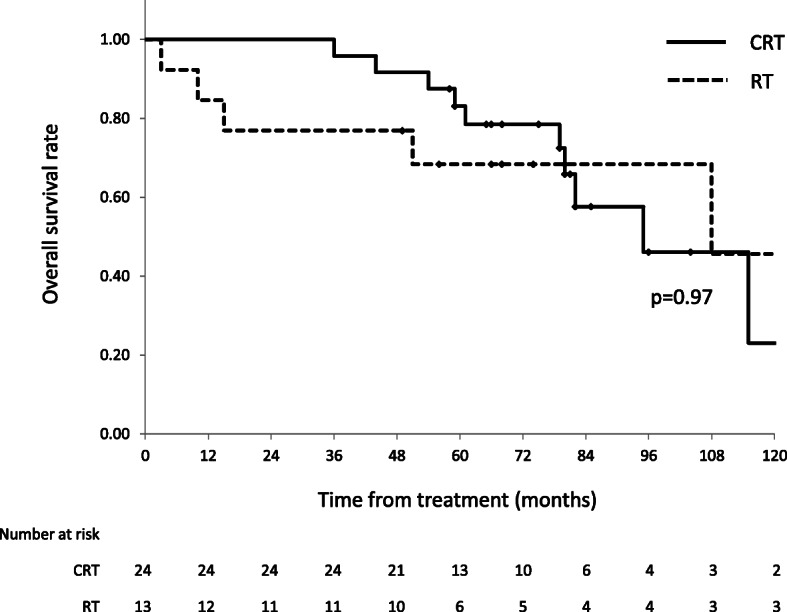
OS rate according to treatment strategy. The 5-year OS rates were 83% in the CRT group (95% CI, 68–98%) and 68% in the RT alone group (95% CI, 43–94%). OS: overall survival; CRT, chemoradiotherapy; CI: confidence interval; RT: radiotherapy

The median follow-up time was 74 months (range, 3–212 months) for all patients and 75 months (range, 49–209 months) for survivors. The 5-year OS and PFS rates were 78% (95% confidence interval [CI], 64–91%) and 64% (95%, CI 49–80%), respectively (Fig. 1). There was no significant difference in OS between chemoradiotherapy (CRT) and RT alone (Fig. 2). Regarding failures, no local or regional recurrences were observed. Distant failures were observed in two patients (5%): distant lymph node metastases in one patient treated by CCRT and carcinomatous pleurisy in one patient treated by EBRT and IBT. The patient who suffered distant lymph node metastasis in the supraclavicular fossa and abdominal para-aortic region (both sites were outside the RT field) at 45 months after CCRT underwent salvage surgery and postoperative CCRT. He died of arrhythmia and heart failure at 61 months after CCRT, with no evidence of recurrence of esophageal cancer after salvage treatment.

### Metachronous esophageal cancer and other malignancies

**Fig. 3:**
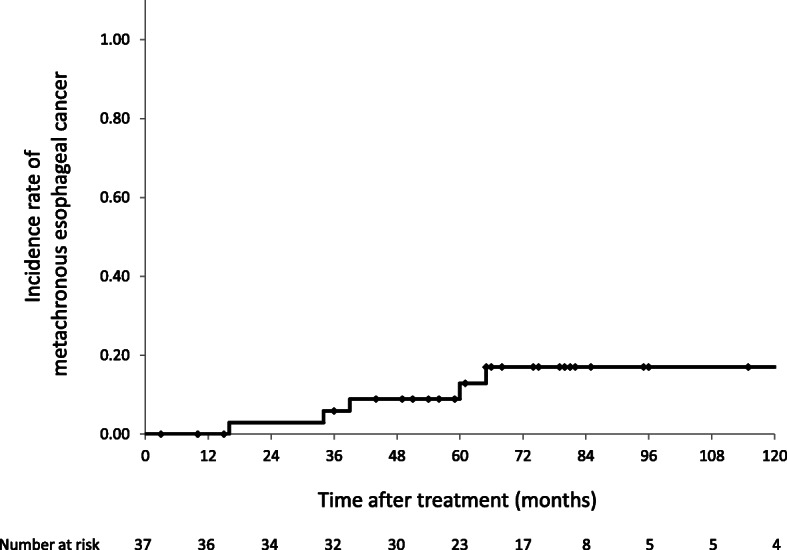
Incidence rate of metachronous tumor. The 5-year incidence rate of metachronous tumors was 13% (95% confidence interval, 0.9–24.8%)

Metachronous esophageal cancer was observed in seven patients (19%). The median duration from the end of treatment to diagnosis of the metachronous tumor was 60 months (range, 16–203 months). The 5-year incidence rate of metachronous tumors was 13% (95% CI, 0.9–24.8) (Fig. 3). The relation between metachronous esophageal cancer and irradiation field was following: within boost field in two patients, within ENI field in three patients and outside the field in two patients. As both cases of metachronous esophageal cancer within the boost field were away from the excision scar, we diagnosed them as newly occurred esophageal cancer and not recurrence. Five patients were salvaged by ER. One patient with a submucosal lesion concurrently suffered leukemia and received best supportive care because of the poor performance status and older age. One patient refused treatment and died of esophageal cancer.

Double cancer after initial treatment was observed in 15 patients (41%). One patient had three malignancies and four patients had two malignancies: head and neck cancer in six patients, gastric cancer in five patients, colorectal cancer in two patients, hepatocellular carcinoma in two patients, malignant lymphoma in two patients, and single cases each of lung cancer, bile duct carcinoma, pancreatic carcinoma, and leukemia. All patients with gastric cancer were diagnosed as superficial carcinoma and salvaged by ER.

### Causes of death

At the time of the last follow-up, 16 of 37 patients had died. One patient with carcinomatous pleurisy died of esophageal cancer. One patient died of metachronous esophageal cancer that occurred 17 years after the initial treatment. Eight patients died of other malignancies, including head and neck cancer in three patients and single cases each of bile duct carcinoma, pancreatic carcinoma, hepatocellular carcinoma, malignant lymphoma, and leukemia. All cases were double cancer occurring after RT. Six patients died of other causes: single cases each of pneumonia, heart failure, respiratory failure, renal failure, senility, and traffic accident.

### Toxicity

The toxicities are summarized in Table 2. Grade 3 or worse acute toxicities of esophagitis, leucopenia, thrombocytopenia, and renal function occurred in two (5%), 12 (32%), two (5%), and one (3%) patient, respectively. Grade 5 acute toxicity was not observed. In patients treated by RT alone, grade 3 or worse acute toxicity was not observed. Grade 2 esophageal stenosis occurred in seven (19%) patients and was significantly higher in patients with tumor circumference equal to or more than 3/4: ≥ 3/4 in six patients and < 3/4 or unknown in one patient (*p* < 0.001). Grade 3 or worse esophageal stenosis was not observed. Grade 2 pericardial effusion was observed in 13 (35%) patients. CTCAE v4.0 defines grade 2 pericardial effusion as asymptomatic pericardial effusion. Thus, slight pericardial effusion was considered grade 2 and most grade 2 cases in the present study were a small amount of pericardial effusion. Grade 3 or worse late toxicities of pericardial effusion, ischemic heart disease, and arrhythmia were observed in two (5%), one (3%), and four (11%) patients, respectively. One patient with grade 5 arrhythmia had a history of dilated cardiomyopathy before receiving CRT and was judged medically inoperable because of cardiac dysfunction. Although the causal relationship between arrhythmia and RT was unknown in this case, it was categorized as grade 5 toxicity because we could not rule out a potential relationship. Although the univariate analyses did not show any significant factor to be associated with late cardiac toxicities, grade 3 or worse toxicities tended to be more frequent in patients treated by RT alone or anterior-posterior opposed field (Table 3).

**Table 2:** Toxicities

Toxicities	No. of patients (%)			
	Grade 2	Grade 3	Grade 4	Grade 5
Acute				
Esophagitis	2 (5)	2 (5)	0	0
Nausea	1 (3)	0	0	0
Leucopenia	12 (32)	12 (32)	0	0
Thrombocytopenia	3 (8)	1 (3)	1 (3)	0
Renal function	3 (8)	1 (3)	0	0
Late				
Esophageal stenosis	7 (19)	0	0	0
Pleural effusion	4 (11)	0	0	0
Radiation pneumonitis	1 (3)	0	0	0
Pericardial effusion	13 (35)	1 (3)	1 (3)	0
Ischemic heart disease	0	1 (3)	0	0
Arrhythmia	0	2 (5)	1 (3)	1 (3)

**Table 3:** Late toxicities: cardiac toxicities

Characteristics	n	≥ Grade 2		≥ Grade 3	
		n (%)	*p-*value	n (%)	*p*-value
Age (years)					
≤ 67	19	9 (47)	1.00	4 (21)	0.66
> 67	18	9 (50)		2 (11)	
Tumor length (cm)					
≤ 3	22	9 (41)	0.32	4 (18)	1.00
> 3	15	9 (60)		2 (13)	
Tumor location					
Ce-Ut	6	1 (17)	0.18	0 (0)	0.56
Mt-EGJ	31	17 (55)		6 (19)	
Treatment					
CRT	24	12 (50)	1.00	2 (8)	0.16
RT alone	13	6 (46)		4 (30)	
History of heart disease					
Yes	9	6 (67)	0.27	2 (22)	0.62
No	28	12 (43)		4 (14)	
Initial field					
Anterior-posterior	20	11 (55)	0.75	5 (25)	0.19
Multiple	17	8 (47)		1 (6)	

*EGJ* Esophagogastric junction

## Discussion

Advances in endoscopic equipment have contributed to the increased detection of early-stage esophageal carcinoma; in addition, the number of patients with superficial esophageal cancer treated by ER has also increased. According to the Registry of Esophageal Carcinomas in Japan, superficial esophageal cancer accounted for 22.7% of esophageal cancer patients treated in 2001 and 33.4% in 2011. In the same reports, 11.3 and 17.2% of patients with esophageal cancer were treated with ER in 2001 and 2011, respectively [1, 17]. Difficulty in the accurate diagnosis of the invasion depth by endoscopic examination and recently expanded indications for ESD, such as tumor of entire circumference or MM invasion, has caused an increase in the number of non-curative resections requiring additional treatment. This study retrospectively analyzed the efficacy and toxicity of salvage RT for superficial esophageal cancer with non-curative ER. The 5-year OS rate was 78% (95% CI, 64–91%), and only one patient died of primary esophageal cancer. Several reports have shown the 5-year OS rate of stage I esophageal cancer patients treated with esophagectomy of approximately 64–78% [7, 8, 18]. Our results and recent reports of definitive CRT for superficial esophageal cancer were comparable to these results [12–14]. Moreover, RT is less invasive compared to esophagectomy and has obvious advantages for organ preservation. Although previous reports showed RT alone was inferior to CRT in esophageal cancer, we did not observe a significant difference in OS between CRT and RT alone [12, 14, 19, 20]. We previously reported the long-term outcome of IBT in combination with EBRT for superficial esophageal cancer [11]. In that report, the most common failure pattern was the primary site, and regional lymph node metastasis tended to occur more frequently in submucosal cases. Resection of the primary tumor by ER may have contributed to local control in the present study. In addition, our results showed good regional control in cases of RT alone. Our cases were clinically judged to be suitable for treatment with ER. Hence, the risk of regional lymph node metastasis might be lower than that of esophageal cancer clinically diagnosed with submucosal invasion with no indications for ER. Although CRT is standard therapy for esophageal cancer, our results suggest that RT alone after non-curative ER might be a worthwhile, less toxic treatment option for patients who are difficult to administer chemotherapy.

The optimal radiation field and efficacy of ENI for esophageal cancer remain controversial. Although a recent meta-analysis did not indicate the effectiveness of ENI, cases of locally advanced esophageal cancer were mainly included [21]. Moreover, the most common failure pattern was local failure in advanced cases [22]. These findings suggest that poor local control may affect the limited contribution of ENI. Furthermore, no multi-center randomized phase III trials have evaluated the effectiveness of ENI for early esophageal cancer. In this study, all patients received ENI and none experienced regional lymph node metastasis, even though they were at risk of subsequent lymph node metastasis. We believe that the use of ENI contributed to this high regional control rate. Early esophageal cancer can achieve high local control rate by CRT compared with advanced cases and higher local control could be expected after ER. Thus, the effectiveness of ENI for early esophageal cancer should be investigated focused on this cohort. The concern of RT with ENI is the increased risk of severe cardiopulmonary toxicities. In our study, grade 3 or worth cardiopulmonary toxicities were observed in six patients (16%), an occurrence rate we considered to be acceptable. However, it is important to reduce the irradiation dose to the heart as much as possible. Although we did not identify any factor associated with late cardiac toxicities, five of them were treated by anterior-posterior field. Recently, the use of multi-portal beams to reduce cardiopulmonary toxicities has become standard in esophageal cancer radiotherapy. Recent advanced techniques such as IMRT or proton therapy have the potential to reduce cardiopulmonary toxicities. Lin et al. reported the efficacy of IMRT for esophageal cancer patients. They observed a significantly higher cumulative incidence of cardiac-related deaths in the 3D-CRT group compared to that in the IMRT group [23]. In addition, they also reported that the use of IMRT may be associated with reduced all-cause, cardiac-related, and other-cause mortality in elderly patients with esophageal cancer [24]. Moreover, proton therapy can improve target coverage while reducing the irradiation dose to the surrounding normal tissue compared to photon therapy and proton therapy is expected to achieve high locoregional control and reduce RT-induced toxicity [25, 26].

One concern regarding toxicity in this treatment strategy is esophageal stenosis. In our study, although grade 2 esophageal stenosis was observed in seven patients (19%), they were manageable and severe stenosis was not observed. The reported occurrence rates of esophageal stenosis after ER were 68–94% for tumor circumferences ≥3/4 and were significantly higher in cases with tumor circumference < 3/4 [27–29]. In our study, a tumor circumference ≥ 3/4 was also significantly associated with esophageal stenosis: ≥ 3/4 in six patients and < 3/4 or unknown in one patient (*p* < 0.001). Therefore, the indication for ER should be judged carefully, especially in cases with tumor circumference ≥ 3/4 and high probability for the requirement of additional treatment at clinical diagnosis.

On the basis of the results of the Japan Clinical Oncology Group (JCOG) trial, 60 Gy is considered standard treatment for both locally advanced and early stage esophageal cancer in Japan [14, 30]. Owing to the lack of the evidence about RT after non-curative ER, we used the same protocol of definitive CRT in the current study. The JCOG 0508 trial is a phase II trial that evaluated the use of the combined ER and CRT for clinical stage I esophageal cancer. In JCOG 0508 trial, patients with clinical stage I submucosal (cSM1–2) esophageal cancer received diagnostic ER and selective CRT based on the histological status. Group A, defined as pathological mucosal invasion with negative resection margin and no LVI, received no additional treatment, while Group B, with pathological SM invasion with negative resection margin or pathological mucosal invasion with LVI, received prophylactic CRT (41.4 Gy) and Group C, with pathological SM invasion with positive resection margin, received definitive CRT (50.4 Gy). The 3-year OS rates were 90.7% for Group B and 92.6% in all patients [31]. This result was comparable to that of surgery or CRT for clinical stage I esophageal cancer. Thus, a high dose such as 60 Gy, might not be needed for esophageal cancer after ER.

Metachronous esophageal cancer is a grave issue in patients who have undergone organ preservation treatment for esophageal cancer. The incidence rate of metachronous cancer after ER are 13–14.6% [32, 33]. In the present study, seven patients (19%) experienced metachronous cancer and the 5-year incidence rate was 13%. Five of the seven patients were successfully salvaged by ER. Therefore, the detection of metachronous cancers as superficial lesions by close endoscopic observation is important.

Esophageal cancer patients are at high risk for other malignancies such as head and neck, gastrointestinal, or lung cancers. In our cases, 15 patients (41%) experienced other malignancy after RT, and the most common cause of death was other malignancies. Early detection of other malignancies is also important after RT since patients with superficial esophageal cancer can expect long-term prognosis.

Our study was limited by its retrospective nature, small number of patients, and variety of RT methods and chemotherapeutic regimens. However, as there are few reports on the long-term results of salvage RT after non-curative ER for superficial esophageal cancer, we think that the results of this study are of great significance. Diagnostic ER and selective CRT based on histological status are likely to become the standard treatment strategy for submucosal esophageal cancer instead of surgery. Our results suggest that RT after ER is a safe and effective treatment while preserving organs and that a longer follow-up is required for the early detection of metachronous esophageal cancer and other malignancies.

## Conclusion

Salvage radiotherapy after non-curative esophageal endoscopic resection is an effective treatment as an organ preservation strategy. Although muscularis mucosae and submucosal cancer have high risks of lymph node metastasis, our results suggest that ENI contributes to reduced regional node metastases. Early detection of metachronous esophageal cancer and other malignancies is important for survivors.

## Data Availability

Data in the manuscript are available by contacting the corresponding author.

## Abbreviations

CCRT: Concurrent chemoradiotherapy
CI: Confidence interval
CRT: Chemoradiotherapy
CT: Computed tomography
CTV: Clinical target volume
EBRT: External beam radiotherapy
EGJ: Esophagogastric junction
ENI: Elective nodal irradiation
ER: Endoscopic resection
ESD: Endoscopic submucosal resection
IBT: Intraluminal brachytherapy
IHD: Ischemic heart disease
IMRT: Intensity-modulated radiotherapy
JCOG: Japan Clinical Oncology Group
LN: Lymph nodes
MM: Muscularis mucosae
SM: Submucosal layer
OS: Overall survival
PFS: Progression-free survival
